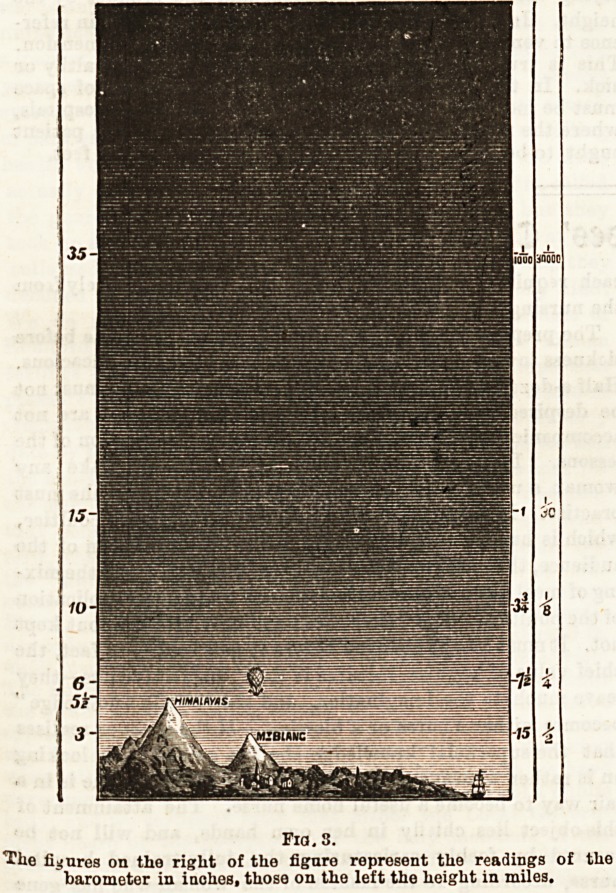# "The Hospital" Nursing Mirror

**Published:** 1896-05-02

**Authors:** 


*The Hospital*, May 2, 1896. *Extra Supplement.*

### "The Hospital" Nursing Mirror.

Being the Extra Nursing Supplement of "The Hospital" Newspaper.

[Contributions for this Supplement should be addressed to the Editor, The Hospital, 428, Strand, London, W.C., and should have the word "Nursing" plainly written in left-hand top corner of the envelope.]

### News from the Nursing World.

### THE QUEEN'S RETURN.

The Queen's loyal subjects will all rejoice that her stay at Cimiez should have been favoured with the best of weather, and that Her Majesty has been able to enjoy it to the full, taking daily drives, and usually breakfasting in a tent in the beautiful garden of the Hotel Cimiez. At the end of this week the Queen will be once more home again at Windsor, accompanied by the Princess Beatrice and her children.

### INVALIDS AT DAVOS.

An appeal for funds to continue the support of the Invalids' Home at Davos has recently appeared in the *Times*. For the past eleven years this home, which is the only English institution in the Alps for consumptive patients who are not able to afford the heavy cost of hotel living, has been carried on by private effort and private financing. Now it has become necessary, if a very useful institution is not to be altogether given up, to establish it on public lines, and to purchase the house and furniture. This is to be done at a cost of £2,750. About £1,500 has been received in consequence of friendly efforts, and one who is interested in the home and its work has promised to give £250 on condition that the remaining £1,000 be provided at once. For the sake of the many people to whom admission to the Invalids' Home may in the future mean a new lease of life, it is much to be hoped that the response received will be prompt and generous. Contributions to be sent to the bankers of the fund, Williams, Deacon, and Co., Manchester and Salford Bank (Limited), 20, Birchin Lane, E.C.

### NINETEENTH CENTURY CIVILISATION.

The accommodation provided by the Local Government Board in their modern workhouse infirmaries is, as a rule, all that can be wished, and, in certain instances, infinitely better than at many voluntary hospitals. But in some workhouses discomfort reigns supreme, as at Aston, Birmingham, where the public has learnt, with a shudder of disgust, that "the nurses had to use a room for assembling where convalescent men were smoking and spitting, and where there was not the least bit of comfort." Mr. Cooke, the Guardian who drew attention to this deplorable state of affairs at the last Board meeting, gave notice to move at the next meeting, "That the General Purposes Committee be instructed to prepare a scheme for the provision of proper accommodation for the nurses." It is to be hoped that, in this most urgent case, the needful steps will be taken without any of the opposition which usually attends any improvement in workhouse administration.

### PHYSIOLOGY v. WOMANLINESS.

Miss Frances Power Cobbe has been quite horrified by the idea that, in the course of physiology classes, schoolgirls are allowed to dissect dead rabbits. It is, she thinks, "emphatically the lower education of women, namely, that which is best suited to deprive them of womanliness." The teaching of physiology is, to Miss Cobbe's mind, "part of the large aim of a certain powerful party to familiarise the public mind with the idea of vivisection, to inspire interest in the results of such 'research,' and to transform natural horror of cruelty into the morbid fascination which the sight of blood and mutilation manifestly possesses for their unhappy selves." Surely prejudice can go no further! Are nurses examples of unwomanly women, one may wonder, for as the study of the human body is a part of their training, they might be supposed to show the first signs of the deterioration to which "the coarsening influence of such studies" leads, according to Miss Cobbe. We may be thankful that such an hysterical cry as this is likely to find few supporters in these days of greater practical common sense, and it is to be hoped that soon it will be the rule for every woman, from the highest to the lowest, to know something more than they do at present of the marvellous structure of the human body—a knowledge which can only be to the immense advantage of the whole human race.

### THE ADVANTAGE OF A TRAINED NURSE.

The appointment of a trained nurse at the Michaelstown Union Infirmary in Ireland has led to the exposure of a most unsatisfactory state of affairs. The nurse resigned stating that she could not prevent the cruelty exercised towards the patients by one of the nurses, and preferred to leave in consequence. The Guardians expressed themselves grateful to the nurse for exposing the matter, and have appealed to the Local Government Board to hold a thorough investigation. The patients corroborate all the nurse's statements.

### WORKHOUSE NURSING AT YARMOUTH.

The Board of Guardians at Yarmouth are about to make certain needful alterations in the nursing arrangements at the infirmary. A report from the Visiting Committee, read at a recent Board meeting, contained recommendations to the effect that the inmate wardswomen should continue to perform the domestic work of the infirmary; that probationers should assist in the nursing of the sick and infirm paupers; and that two nurses be advertised for. Mrs. Leach, one of the Guardians, congratulated the committee on having arrived at the conclusion that nursing was a profession necessitating careful training, and remarked that while she was glad the nursing was to be done by trained persons she regretted that "the salary was limited to £15 a year for each nurse." It is manifestly impossible to secure "trained" nurses for such a pittance, and if the training of probationers is to be entrusted to "nurses" whose qualifications (*vide* advertisement) need only be competency "to assist generally in nursing the inmates of the infirmary wards" the results are likely to be anything but satisfactory.

### IRISH WORKHOUSE REFORM.

A conference, with the object of forming an association for the furtherance of reform in Irish workhouses, was held at St. Martin's Town Hall on Wednesday. The chair was taken by Mr. Charles S. Roundell, and the meeting was addressed by Dr. Hamilton Moorhead, Miss Catherine J. Wood, Dr. Jacob, and other speakers, a resolution being finally moved by the Hon. Horace Plunket, M.P. Dr. Jacob's stirring words, coming after the graphic details given by Miss Wood and Dr. Moorhead, must surely have roused the audience to a conviction that something must be done, and that speedily, to remove a condition of affairs which, in Dr. Jacob's words, is atrocious in a civilised and Christian country. A report of the proceedings will be given next week.

### WORK TO BE DONE.

We are asked by Miss Gill, secretary to the Workhouse Infirmary Nursing Association, to remind those who are contemplating entering the nursing profession of the important work that is to be done in the field of workhouse nursing, for which women of superior education and intelligence are much needed. To successfully carry on the work of reform a special type of nurse is needed—to battle with ignorance and put up with difficulties which are never encountered in ordinary hospital work; tact and forbearance, and a trained mind and temper are required as much as a knowledge of the theory and practice of nursing. It is still pioneer work in workhouse nursing, and often very disheartening, but, after all, to the pioneers belong the glory, and it is certain that the universal substitution of trained service for pauper help will be tremendously assisted by the brave efforts of those who lead the way. Applications from well-educated women, ready to sink themselves in the work they have to do, will be gladly welcomed by Miss Gill at the office, 6, Adam Street, Strand, W.C., every afternoon except Saturday, between half-past two and half-past five.

### NURSES' INSTITUTION, SALISBURY.

At the recent annual meeting the committee of the Salisbury Nurses' Institution reported a satisfactory year's work. The nurses had been much in demand, and the staff had been increased by two. In moving the adoption of the report, the chairman, Mr. A. Buckley, spoke of the desirability of giving the nurses two years' training at the infirmary instead of one as at present, and his views on the subject were fully endorsed by Mr. Luckham, one of the medical officers. Financial considerations are said to stand in the way of this most desirable step, but the Salisbury Institute has many friends, and if they can be made to recognise that its future well-being depends upon the employment of really efficient nurses, to make which one year in a hospital is certainly not adequate, there should be no difficulty in carrying out Mr. Buckley's wise recommendation.

### WHAT WOMEN CAN DO FOR WOMEN.

In support of the scheme initiated by the women students of Edinburgh University on behalf of women workers in shops and warehouses, a deputation of ladies was recently received by the Town Council of Edinburgh, to urge their consideration of a recommendation which is being addressed to the "merchants, warehousemen, and shopkeepers in Edinburgh," in favour of providing seats for women employés. The Council, on the motion of Lord Provost McDonald, unanimously agreed to use their influence in support of the movement. Edinburgh is setting a good example in this respect, and it is much to be wished that some strong effort should be made in London in the same direction. It is a disgrace to the women who spend hours of every week in London shops, that in so many well-known and popular establishments to-day no seats are allowed to the assistants who serve in them. Statistics and reports on female labour have pointed out again and again how terrible are the after-results to health of this long and unnecessary standing; and still year after year goes by and the women with leisure, money, and influence, who crowd the shops, pursue their bargaining in heartless disregard of the cruelties daily practised on those other women who minister to their wants across the counter, and who cannot themselves protest without facing a possible loss of employment. "Shopping women" too often deserve the reflection cast upon them by the assistant who replied to a clergyman's representation of Christ's life of hard work, "Yes, but there were only men in the carpenter's shop. He didn't have to serve *women* all day over a counter."

### SMART BUT NOT SERVICEABLE.

"You have secured an excellent nurse," said the surgeon, "she helped us admirably at the operation." "Yes," replied the general practitioner, "she's quite up-to-date as an assistant, but I don't know anything about her nursing." The two friends met again a few weeks later. "How did that nurse turn out?" asked the surgeon who had operated. The general practitioner shook his head. "I shall never employ her again," he said, "she may be smart enough at handing sponges or naming instruments, but she doesn't know how to keep a patient comfortable, nor a bed free from crumbs."

### SHORT ITEMS.

The Barry Dictrict Nursing Association have received a donation of £10 from the Welsh Rugby Football Union.—The number of the Naval Nursing Sisters has this year been increased by two, the total staff now numbering three head sisters and nineteen nursing sisters. For the first time a sister has been stationed at the Naval Cadets' Hospital, Dartmouth.—The Troon District Nursing Association is to be congratulated on beginning a new year with a surplus of £62 3s. 2d. The Duchess of Portland was re-elected president at the annual meeting.—The Mayor of Blackburn has convened a meeting to "consider the advisability of establishing a scheme for district nursing in Blackburn on similar lines to the successful organisations which have been working for the last few years in Bolton, Darwen, and other towns."—The committee of the Barry Nursing Association have elected Miss Sykes, late superintendent of one of Lady Roberts' hospitals in India, to the post of lady superintendent, in succession to Miss Amy Evans.—The twentieth annual meeting of the Metropolitan and National Nursing Association will, at the invitation of the Duke of Westminster, be held at Grosvenor House on Tuesday, the 5th of May, at three o'clock.—An article in the *English Illustrated Magazine* for May, on the London Hospital "Receiving Room," will be read with interest by all "Londoners." Amongst the sketches is a faithful one of "Fewell," the night porter, a familiar figure for twenty years past.

### Hygiene: For Nurses.

By JOHN GLAISTER, M.D., F.F.P.S.G., D.P.H.Camb., Professor of Forensic Medicine and Public Health, St. Mungo's College, Glasgow, &c.

### IV.—AIR IN RELATION TO HEALTH—ITS COMPOSITION AND COMMON IMPURITIES—CUBIC AND SUPERFICIAL SPACE.

AIR is one of the prime necessities of life. Without it, animal and vegetable life could not exist, and combustion could not go on either in its chemical or physical aspects. All animals, whether they live in the ocean of air which surrounds the planet, or in the ocean of water which encompasses the land of which it is composed, demand air in one form or another for their existence. All animals may be divided into two main classes, viz., those which inhale air *directly* from the atmosphere, and those which inhale air *indirectly*. To the latter class belong the myriads of

FIG. 3.
The figures on the right of the figure represent the readings of the barometer in inches, those on the left the height in miles.

animals which live in water. They obtain their supply from the air which is dissolved in the water, consequently their lives depend upon the efficient aeration of that fluid. A simple experiment with fish in a jar will demonstrate this.

We live at the bottom of an ocean of air. If in imagination we picture ourselves living at the bottom of the sea, where all around is water, we will be better able to estimate our position on the earth, where all around is air. Air, like water, is a fluid, and in several respects obeys the same laws. It possesses weight and elasticity, and is capable of contraction and expansion by cold and heat. By our senses we can easily perceive the weight of water, not so with air. But the latter, like the former, may be weighed in a balance. A cubic foot of dry air at 32 deg. Fahr., and at 30 in. of barometric pressure, weighs 566·85 grains; a similar bulk of water, 61¼ lbs. If it is desired to measure the weight of the atmosphere on a large scale, that is effected by means of the barometer (Gr. *baros*—weight, and *metron*—a measure), or the weight-measure applied to air. When, for instance, the mercury in the barometer at the sea-level stands at 30 in. on the scale, a certain atmospheric weight or pressure is sustained, but if a hill about 2,000 ft. in height is ascended, the mercury will now be found to stand at about 28 in., and the higher the ascent the greater the fall of the mercury becomes. The reason for this is simple. As we ascend, the weight of the atmosphere becomes less, and, in consequence, presses with less force on the mercurial column. Hence it is, that if we could ascend three miles above sea-level, the barometric reading would be found to be but little more than one-half of that at sea-level; or, to a height of five miles—about the height of the highest Himalayan peaks—to a little more than one-quarter of the sea-level reading. Fig. 3 illustrates this.

Atmospheric pressure—which varies from day to day and often from hour to hour—has a determinate influence on the health of the people. It has a direct influence on the "fiery" condition of coal mines; explosions occur more frequently when the pressure is great than when it is light. It is directly associated, also, with weather changes.

Air also possesses elasticity. If a thin glass globe be filled with air at 40 deg. Fahr. and sealed, and the temperature then raised to 100 deg. Fahr., the globe would be shivered into pieces. This is due to expansion of the contained air. Conversely, air may be compressed into smaller bulk; indeed, it has been recently liquefied so that it could be poured from one vessel to another. The usefulness of the aneroid barometer depends upon this phenomenon of expansion and contraction of air.

COMPOSITION OF AIR.—Pure air consists of a mechanical mixture of certain gases. It is not a chemical mixture. The difference between the two is very distinct. In a mechanical mixture the component parts retain their individual identity, and do not form new compounds. In a chemical mixture the constituent parts lose their identity, and become transformed into new compounds. However intimately we grind together salt and sugar, they will remain for all time as a mechanical mixture of salt and sugar; and the same would happen if they they were dissolved in water. If, on the other hand, we put some clear lime-water into a bottle, and breathe into it, the water will now become milky, owing to a new substance having been formed by the union of the carbonic acid of the breath and the lime-water. This would be a chemical mixture.

The gases of which air, in its purest form, is composed, are oxygen, ozone, nitrogen, carbonic acid, and watery vapour in a gaseous form. In 1,000 volumes of such air oxygen = 209·6 + nitrogen = 790·0 + carbonic acid gas ·4 = 1,000. The amount of watery vapour and ozone are very variable. Volumes may be called pounds, ounces, gallons, or any other measure or weight, since these proportions are relative in any amount in any measure or weight. By shifting the decimal point one place to the left, percentages are obtained. Oxygen is the vital constituent of air. By it combustion in any form is sustained, and it is the source by which the blood in the lungs is purified. The act of breathing consists essentially of inspiration, *i.e.*, the taking-in of air, and of expiration, *i.e.*, the out-putting of air. In the former we inhale fresh air, in the latter we exhale foul air. The composition of expired air is therefore very different from that of fresh air. In 1,000 volumes of it, oxygen = 169·2 +

nitrogen = 790·4 + carbonic acid gas = 40·4; together with watery vapour and organic matter. The nitrogen serves the useful purpose of diluting the oxygen for the needs of the body. It is an important agent, also, in plant nourishment, being, in thunderstorms, converted into such a form as to enable plants to assimilate it. Carbonic acid gas may be deemed, so far as man is concerned, a normal adulterant or impurity of air, but it also serves a useful purpose as plant-food, as during sunlight, the green-colouring matter of plants splits it up, absorbs the carbon, and liberates the oxygen. Probably, therefore, for plants it is an essential atmospheric constituent.

IMPURITIES OF AIR.—These may be divided into two main classes, viz. (1), those that are particulate, and (2) those that are gaseous. The cause which chiefly determines the amount of both is the congregation of persons in a limited area. In populous places, the former class mainly consists of unconsumed particles of coal-soot, débris from the tear and wear of the bodies of men and animals, of the streets, and from industrial operations, of both inorganic and organic composition; and micro-organisms. The second class is composed chiefly of the gaseous products of combustion and of chemical works —these being mainly compounds of ammonia and sulphur of different chemical compositions. Hundreds of thousands of tons of these gases are computed to be given off annually into the atmospheres of large cities. Carbonic acid gas is also largely increased in these circumstances. In foggy weather, in Manchester, London, or Glasgow, this gas may be increased from four to seven parts per 10,000 of air. The air of a populous place is, therefore, notably more impure than that of the open country or the sea. In like manner the air of an occupied room becomes impure by increase of carbonic acid gas, watery vapour, and organic matter. In schools, for example, this gas may be found present from five to ten times more plentifully than in pure air, and organic matter twenty times more.

SPACE IN REFERENCE TO AIR SUPPLY.—By superficial space is meant space multiplied into two dimensions, viz., the length by the breadth; by cubic space, into three dimensions, viz., length by breadth, by height, and thickness. Feet multiplied by feet equals square feet, and feet × feet × feet equals cubic feet. Both measurements are of great importance in reference to air supply, the one as much as the other. Superficial space in reference to a room is often called floor-space or floorage. The cubic space of a room is obtained by multiplying the length by the breadth, and the product by the height. In considering the dimensions of any room in reference to ventilation, regard must be had to each dimension. This is true of rooms whether occupied by the healthy or sick. In the latter case, however, each dimensions of space must be increased, and particularly so in infectious hospitals, where the minimum amount of superficial space per patient ought to be 100 square feet, and of cubic space, 1,200 feet.

### Trained Nurses' Clinic.

### III.—NURSING AT HOME.

### UNTRAINED NURSES.

THE untrained or partially-trained women who represent themselves as professional nurses, without having the slightest claim to be so considered, do not come within the scope of this article. The untrained nurses herein referred to are rather the women in every class of life who are called upon to attend on many sick beds. Some of them have had so much experience of this sort that they have acquired practical knowledge equivalent to a great deal of so-called training. These home nurses have none of the pretensions of half-trained women, and are generally those members of the family whom a process of natural selection has pointed out as the best all round, capable, and intelligent of their sex.

These home nurses often make light of the difficulties they have to contend with, and endeavour in a quiet way to supplement their knowledge by books, by the doctor's instructions, and by hints from those whom they think are wiser than themselves. The home nurse suffers a good deal sometimes from the amount and diversity of the advice she receives, and it is often hard for her to avoid offending those with whose instructions it is impossible for her to comply. Difficulties must often arise on account of the lack, in the case of the home nurse, of the authority which is assumed instinctively by the trained nurse, and which is rarely questioned by the patients of the latter. The relation who devotes herself without fee or reward to her own sick is often severely criticised, and her authority frequently set at naught.

Now of all the possessions which give power to those who own them, knowledge ranks first, and hence every woman who aspires to nurse the members of her own family should acquire the needful knowledge. The authority of the trained nurse depends entirely on her knowing more than those she serves, and if she betrays inefficiency she has no longer any weight with her patients or their friends.

The same thing happens with the home nurse if she displays sufficient ignorance to shake the patient's confidence. It would therefore be well if everyone learnt as much as possible about ordinary everyday illnesses and the special care which each requires. Of course this should be done entirely from the nursing, not from the medical point of view.

The preparation for home nursing should be made before sickness invades the household, if it is to be really efficacious. Half-a-dozen popular lectures on first aid in nursing must not be despised; but they are of little value if they are not accompanied and followed up by a practical application of the lessons. Listening and looking on will never make any woman a useful home nurse. She must reflect and she must practise. If the lecturer merely makes a neat little poultice, which is handed round for the admiring inspection of the audience, the latter may be excused for forgetting that the mixing of meal and water must be followed by the due application of the poultice. The latter must not only be made, but kept hot. It must also be changed before it gets cold. In fact, the chief value of popular lectures is their suggestiveness—they leave much to the imagination, and the "little knowledge" becomes either a curse or a blessing. If the listener realises that the superficial knowledge she has gained from looking on is rather a means to an end than the end itself, she is in a fair way to become a useful home nurse. The attainment of this object lies chiefly in her own hands, and will not be secured by feebly caricaturing the fully-trained hospital nurse, according to the fashion of the woman who has gone in for a little training.

The best work can only be accomplished by practise, and therefore every fragment of technical knowledge which the home nurse acquires should be patiently utilised. She should employ both hands and brains, and she should incessantly study, in the days of health, the tastes and idiosyncrasies of those to whom in sickness it will be her duty to devote herself. The woman who does this is generally capable of giving intelligent and acceptable service in all cases of ordinary illness.

### Wants and Workers.

NURSE MINNIE would be glad to know if there is any society that can help district nurses to dispose of needlework or knitted articles made by the poor in her parish? Address, Nurse Minnie, care of the Editor.

### Poor Law Infirmary Nurses and the Superannuation Bill.

THE Superannuation Bill for poor law officers now before Parliament is most unjust, so far as it affects matrons and nurses working in poor law infirmaries or under the poor law in any capacity. The Bill as drawn is not applicable to nurses and attendants on the sick. It fixes the age of retirement at 65, whereas experience shows that in the case of nurses the superannuation should be so drawn as to enable the nurse to retire at 55, with the option of a voluntary retirement five years earlier on conditions defined in a schedule to the Act.

We imagine there must be more than one or two members of Parliament who sympathize with the hard and often cruel lot of a trained nurse working under the poor law in certain parts of this country. We have a case now before us of a nurse of excellent character and great devotion who laboured on under the Romford Board of Guardians for two and a-half years, having charge of six wards in the building and two infectious wards outside, a most improper arrangement in itself. This nurse had charge of patients, and was only allowed the use of a pauper assistant in each ward, so that at the end of two years and a half her health was shattered, her nervous system undermined, and she was prostrated with an attack of paralysis and mania. This nurse exhibited signs of failing health and symptoms of serious illness long before the crisis actually took place. The medical officer, to his credit, called the guardians' attention to the nurse's condition, but they took no step to help the poor woman, and when she at last collapsed utterly, and her resources were exhausted, they declined to make her any grant or allowance of any kind. We do not know what kind of people the inhabitants of Romford may be, but we imagine they must have human feelings and hearts, and, if this be so, then we hope that pressure may be brought to bear upon the Romford Board of Guardians, or that they may be entirely replaced at an early date by persons with some regard at any rate to justice and the necessity of making adequate nursing arrangements for the sick poor whom they nominally look after. We hear that some improvements have been introduced at Romford, thanks to the appointment of lady guardians, but the slur of gross injustice and cruelty to the nurse in question still rests upon the Romford Board, and must continue to operate to the discredit not only of that Board, but to the ratepayers who elected it, until justice is done to the nurse to whose case we here refer. The nurse at the present time is ruined in health and without the means of maintaining herself at all. Her friends have applied more than once to the Romford Board, and such applications have, we understand, been refused. Speaking with full knowledge, we feel constrained to say that we have never met with a case of grosser cruelty on the part of a public body, and we earnestly hope that either the Local Government Board or the ratepayers or the lady members of the present Board of Guardians at Romford will take up the case of this nurse and see that justice is done to her without further delay.

Unfortunately, should the Poor Law Officers' Superannuation Bill pass as it stands, it is calculated to operate with great harshness so far as the nurses are concerned, and we hope some member of Parliament will give notice of amendments to the effect (1) that the provisions of this Act shall not apply to any female employé charged with the duty of attendance upon the sick employed by the guardians of a union or any other authority to which this Act applies; (2) to amend clause 16 by inserting a proviso that any nurse hereafter to be appointed may signify in writing a desire not to join under the new Act; (3) a new clause, empowering guardians and other authorities to prepare a scheme of federation with the Royal National Pension Fund for Nurses, by which provision may be made for superannuation of nurses and other attendants upon the sick in accordance with the plan adopted by several hospitals and similar institutions. The scheme of federation to provide in what proportions contributions shall be made annually by the guardians and the nurses respectively, each scheme of federation in any question arising between guardians or any other authority empowered by this Act to grant superannuation allowances to any of their nurses or attendants on the sick, to be referred to the Local Government Board, whose decision shall be binding and conclusive.

In making these suggestions we wish to impress upon members of Parliament that, whereas the poor-law medical officers, relieving officers, and others employed by the guardians, may remain in the service up to sixty years of age or even later, it is not possible, in the interests of the poor patients or of the nurses themselves, that nurses should remain until they are sixty years of age or upwards nursing the sick in poor law infirmaries. We are informed that the matrons of poor law infirmaries are unanimous in feeling that the Bill, if passed as it stands, will deal very unjustly with nurses. Every lady guardian throughout the country who is interested in the wise administration and proper nursing of the sick poor should write to the member of Parliament for the district or division in which she resides, urging him to watch the Superannuation Bill for Poor Law officers now before Parliament, and, if necessary, to block it until the provisions for the superannuation of nurses are made adequate to meet the necessities of the case. The scheme of the Royal National Pension Fund for Nurses, of which guardians may readily take advantage by federation, is broad and generous, and provides for the peculiar needs of nurses which the Bill totally ignores. A poor law infirmary matron writes on this point: "The more one thinks of the Poor Law Officers' Superannuation Bill as it stands, the more imperative it seems for the friends of nurses to take prompt action against it. I should be very sorry to do anything to wreck the Bill, still I do think there should be some provisional clauses. I do not think it is sufficient to sit quietly down, as we are advised to do, with the comforting assurance that the guardians will pay the premiums or raise the salaries. In the near future guardians may find themselves compelled to adopt this course in order to get competent nurses, but even if this be the case it cannot be used as an argument in favour of a Bill which I consider for the rising generation of nurses is absolutely useless as well as being unjust, from the fact that the money which may be deducted from the nurses' earnings under the Bill may be absolutely lost to them in certain circumstances without any power of redress of any kind."

Dr. Tanner, M.P., is very fond of blocking Bills, and we would specially call his attention as a medical man to the Superannuation Bill for poor law officers, and would urge him to block it forthwith, pending the introduction of clauses which will protect nurses from the injustice and hardship which they must suffer if it is permitted to pass in its present form.

### Minor Appointments.

PASSMORE EDWARDS COTTAGE HOSPITAL, LISKEARD.—Miss S. M. Yeats has been appointed Nurse at this hospital. She received her training at the South Devon Hospital, Plymouth, having also had one year's experience at the Hospital for Women, Nottingham.

HUDDERSFIELD INFIRMARY.—Miss L. H. Peel has been appointed to the post of Charge Nurse at this infirmary. Miss Peel was trained at the Bootle Borough Hospital, and since has worked as staff nurse and sister at the Birmingham General Hospital. We wish her all success.

WORKHOUSE INFIRMARY, GRAVESEND.—Miss M. E. Bailey has been appointed Head Nurse at this Infirmary. She was trained at the Greenwich Infirmary, and has since worked at the Halstead Workhouse, Essex, and at Faversham Workhouse, Kent.

POPLAR AND STEPNEY SICK ASYLUM.—Miss Elizabeth H. Clements, trained at the Mile End Infirmary, Bancroft Road, E., and Miss Florence Barry, trained at Guy's Hospital, have been appointed Ward Sisters at this institution.

### Everybody's Opinion.

[Correspondence on all subjects is invited, but we cannot in any way be responsible for the opinions expressed by our correspondents. No communications can be entertained if the name and address of the correspondent is not given, or unless one side of the paper only be written on.]

### FEVER NURSING.

"Margaret" writes: Having myself been for a short time in one of the fever hospitals under the M.A.B., I have read with interest the letters inserted on the subject. "Fever Nurse," in your issue of April 25th, says the charge nurses all hold a three years' certificate. This is correct in regard to those who have been appointed of recent years; but there still remain those in the position of charge nurse who have not received their full general training. These were formerly considered sufficiently qualified to do the work, and when the present regulations came into force they still retained their posts. Of course, when they leave their places are filled by three years' nurses, so that in due course all charges at fever hospitals will be on an equal footing. In regard to the other points mentioned by your correspondents, let me fully endorse them. The hours off duty are good, the food is plentiful and well served, and the cubicles are light and airy. There is also a good piano and library, and, in addition, Sunday and daily services are so arranged to suit all nurses, whether on day or night duty, which is a great boon to some of the staff.

### THE DIFFICULTIES OF PROVINCIAL MATRONS.

"Another Provincial Matron" writes: I did not see the letter of a "Provincial Matron," and now perhaps any further comment is out of date; but if not I should like to assure a "Physician to a Provincial Hospital" that her feeling is happily not invariable. But while most fully agreeing with him that she has overstated her case and used unfortunate terms, I should say that workers trained in London, and having been, as I was, sister and matron in London hospitals, are likely to find difficulties in provincial work at first. There is much in it that is new to us; we miss a certain "smartness" which the variety and pressure of London work gives to its nurses; and one's first impression of the general style of the hospital may be that of an apparent want of discipline. But I am sure there are many London-trained nurses who, like myself, work happily and find much good to learn in provincial hospitals, while it is quite possible that our London experience will do our nurses no harm from a teaching point of view. I can honestly say that my present staff of nurses, both as to personal character and *esprit de corps*, quite equal those of either of the three London hospitals with which I have been well acquainted. The personal character of a nursing staff in these days of ample choice of material for training will rest with the matron in the long run, either in London or in the provinces.

### Appointments.

Passmore Edwards Cottage Hospital, Liskeard.—Miss Marrietta Lanyon has been appointed to the post of Nurse Matron at this hospital. She was trained at University College Hospital, and has since worked as sister at the Dulwich Infirmary. She takes many good wishes with her to her new work.

Liverpool Training School and Nurses' Home.—Miss Lucy M. Rae has been appointed Assistant Lady Superintendent of this institution. Miss Rae received her training at Charing Cross Hospital, and takes many good wishes with her to her new work. We congratulate her on her promotion.

Gloucester Temporary Small-pox Hospital.—Miss Edith Walker has been selected from amongst a large number of candidates to fill this post. She was trained at the London Hospital, where she afterwards held the position of matron's assistant for nine years, only leaving, in 1892, to the great regret of her fellow-workers, to take up the important work of organising the nursing department at the new Mill Road Infirmary, Liverpool, which she accomplished with most marked success. The experience there gained will be invaluable in helping her to carry out a difficult task at Gloucester, and the authorities are to be congratulated on having secured Miss Walker's services.

### Death in Our Ranks.

Great sorrow has been caused at the Royal United Hospital, Bath, by the death of Nurse Margaret Evans, which occurred on April 12th from typhoid fever, contracted while nursing a patient. Nurse Evans entered the hospital in 1891, and after receiving her certificate remained on the private nursing staff, awaiting a vacancy among the charge nurses in the hospital. She was senior nurse on the roll for promotion when she died. She was buried at Gilfillan, South Wales, on April 17th, and at the same hour a memorial service was held in the hospital chapel, largely attended by members of the committee and medical and nursing staffs. In a short address the President, the Rev. E. Handley, M.A., spoke of the bright example left by Nurse Evans. "It was not only," he said, "her professional skill but also the warmth of her heart, her deep sympathy, her bright, kindly manner, that endeared her to her patients, and to all with whom she came in contact. Truly she had learned how high and noble is the office of a nurse which combines her skill with the heart of a truly Christian woman."

### Where to Go.

Workhouse Infirmary Nursing Association. — The annual gathering of the Mary Adelaide nurses will be held this year, by kind permission of Earl and Countess Brownlow, at 8, Carlton House Terrace, on May 15th, at eight p.m. The medals and gratuities will be presented to the nurses by Constance, Marchioness of Lothian.

A meeting is to be held at Grosvenor House under the presidency of the Duke of Westminster on May 5th, at three p.m., to receive the twentieth annual report of the Metropolitan and National Nursing Association for providing trained nurses for the sick poor.

### Novelties for Nurses.

### DRESS NOVELTIES.

Of the many improvements and discoveries that have taken place of late years in the textile trade, perhaps none claim so large a share of our attention as the process by which fabrics are rendered rainproof. Goods so prepared undergo chemical treatment which renders the garment when worn impervious to the heaviest shower. In many instances the rain does not even spot, but rolls off like the proverbial drops on a duck's back. For the "Patent Millerain Proof," special merits are claimed in this respect. The great porosity of the cloth, which admits of free ventilation, is another important qualification, which tends to give it the preference in the eyes of the public over the close, heating, rubber proof garments we are accustomed to associate with a wet day. Any fabric, whether silk, cotton, or wool, can be submitted to this process, including velveteen, in which the happiest results have been obtained. We have subjected to severe tests a piece of navy blue serge (so useful to members of the nursing profession) which has come unscathed through all trials, thereby proving the immense value of materials so prepared to nurses, especially those engaged in district work who have to be out in all weathers, and under many and varied conditions. Further particulars may be obtained on application to "Millerain" Croft Mills, Halifax, Yorks.

### Notes and Queries.

**Queries.**

(27) *Training.*—Will you please give me your advice? I am eighteen, and wish to enter a children's hospital as probationer as soon as possible. I am not very well educated, but would that matter if I loved the work? Please let me know the price of "How to Become a Nurse."—*Jimpsey.*

**Answers.**

(27) *Training (Jimpsey).*—Your question has been answered many times in these columns. You will not gain admittance into a children's hospital until you are at least 20 or 21. A "love for the work" is certainly a more needful quality than a superior education, but the theoretical part of a nurse's training of course requires a certain amount of general knowledge. You could profitably employ the time of waiting with a study of simple text books on physiology, &c., and in gaining some knowledge of cookery and domestic work, which would help you materially by and bye if you carry out your intention of becoming a nurse.

## Figures and Tables

**Fig. 3. f1:**